# LncRNA AFAP1-AS1 Promotes the Progression of Colorectal Cancer through miR-195-5p and WISP1

**DOI:** 10.1155/2021/6242798

**Published:** 2021-07-13

**Authors:** Yongsheng Li, Zhongpeng Zhu, Xiaokun Hou, Yongjun Sun

**Affiliations:** ^1^Anal-Colorectal Surgery Department, Shengli Oil Field Central Hospital, Dongying 257000, China; ^2^Oncology Department, Shengli Oil Field Central Hospital, Dongying 257000, China; ^3^Medical Department, Shengli Oil Field Central Hospital, Dongying 257000, China; ^4^Gastrointestinal Surgery Department, Shengli Oil Field Central Hospital, Dongying 257000, China

## Abstract

**Objective:**

Colorectal cancer (CRC) is the most common cancer. But, the molecular mechanisms of CRC progression are not fully understood. This study was conducted to explore how the long noncoding RNA actin filament-associated protein 1-antisense RNA1 (lncRNA AFAP1-AS1) participates in CRC progression through the regulation of microRNA-195-5p (miR-195-5p) and wingless-type inducible signaling pathway protein-1 (WISP1).

**Methods:**

The expressions of AFAP1-AS1, miR-195-5p, and WISP1 were detected by RT-qPCR or western blot. A dual-luciferase assay confirmed the target relationship of AFAP1-AS1, miR-195-5p, and WISP1. Colony formation, wound-healing, and Transwell assays were used to detect the growth, migration, and invasion abilities of cells, respectively.

**Results:**

AFAP1-AS1 and WISP1 expressions were notably increased, and miR-195-5p expression was markedly reduced in CRC. The dual-luciferase assay verified that AFAP1-AS1 could bind to miR-195-5p. AFAP1-AS1 knockdown could inhibit the malignant behavior of CRC cells. miR-195-5p could target and regulate WISP1 expression. Overexpression of WISP1 or miR-195-5p inhibition reversed the inhibition effect of AFAP1-AS1 knockdown on the biological activity of CRC cells.

**Conclusions:**

AFAP1-AS1 knockdown may inhibit the proliferation, migration, and invasion of CRC cells through the miR-195-5p/WISP1 axis.

## 1. Introduction

Colorectal cancer (CRC) is a major cause of morbidity and mortality throughout the world. It accounts for over 9% of all cancer incidences and is the third most common cancer worldwide and the fourth most common cause of death [1]. In recent years, the incidence of CRC tends to be less, but the etiology is not clear. Advanced age, high-fat diet, obesity, smoking, familial polyposis, chronic inflammatory bowel disease, and other factors are considered as high-risk factors [2]. The invasion and metastasis of malignant tumors is often the main cause of treatment failure, and 40% to 50% of patients with CRC die from distant metastasis [3]. Radiotherapy and chemotherapy are used to improve symptoms and the quality of life of patients and prolong survival, but it is still not satisfactory [4]. Therefore, in-depth study on the etiology and pathogenesis of CRC is of great significance for clinical diagnosis and treatment.

LncRNAs, more than 200 nucleotide ncRNAs in length, are abnormally expressed in various types of tumor cells and involved in many malignant tumor biological behaviors [5]. With the in-depth research on lncRNAs, more and more lncRNAs were revealed to have a vital role in CRC pathogenesis and progression. Their biological functions include the regulation of proliferation, apoptosis, tumor resistance, and metastasis of CRC, which have great value in clinical diagnosis and prognosis judgment [6]. AFAP1-AS1, the antisense product of the gene encoding AFAP1, is a lncRNA with a length of 6810 nucleotides located on 4p16.1 of the human genome [7]. Recent studies have found that AFAP1-AS1 is highly expressed in many tumors and involved in the regulation of tumorigenesis and development [8]. Currently, studies on AFAP1-AS1 have shown that AFAP1-AS1 is upregulated in CRC, suggesting that the high expression of AFAP1-AS1 involves CRC occurrence and development [9–11], but the specific mechanism of its involvement in tumor-related processes remains unclear.

As ceRNAs, lncRNAs can bind to miRNAs to affect miRNA target genes expression, thus exerting biological functions [5]. Bioinformatics software predicted that AFAP1-AS1 targets miR-195-5p and WISP1 is a miR-195-5p target gene. miR-195-5p was downregulated in CRC, and the overexpression of miR-195-5p obviously hampered the malignant behavior of CRC cells [12]. Wu et al. showed that the expression of WISP1 was increased in CRC tissues, and WISP1 downregulation could effectively hinder the cell proliferation and migration and tumor growth, providing a new target for CRC treatment [13]. Whether AFAP1-AS1 can affect the biological behavior of CRC cells through regulating miR-195-5p/WISP1 is still unknown. This study mainly explored the effects of AFAP1-AS1 on CRC progression and whether it plays a role by regulating miR-195-5p/WISP1, thus providing a target for molecular therapy of CRC.

## 2. Patients and Methods

### 2.1. Clinical Samples and Data Collection

Patients with CRC who underwent surgical treatment at Shengli Oil Field Central Hospital, Dongying, China, from January 2019 to October 2020, were selected as the research subjects. 26 pairs of CRC and matching paracancerous tissue samples were included. All samples were frozen in liquid nitrogen and stored at −80°C. This study was approved by the ethics committee of Shengli Oil Field Central Hospital. Inclusion criteria: (1) patients had not received any other form of antitumor therapy; (2) all patients had undergone surgery for the first time; (3) surgical resection was obtained, and the CRC tissue and paracancerous tissue were confirmed by pathologists; (4) complete clinicopathological data and follow-up data were obtained; and (5) the patient had no mental disorders. Exclusion criteria: (1) distant organ metastasis; (2) complicated with other types of tumors; (3) patients with severe organic diseases; and (4) ulcerative colitis, acute or chronic gastrointestinal inflammation, and autoimmune diseases. All patients signed informed consent.

### 2.2. Cell Culture and Transfections

Human normal colon cell lines NCM460 and CRC cell lines (SW480, SW620, HCT-8, and HCT-116) were purchased from the Cell Resource Center, Chinese Academy of Sciences (Shanghai, China). The abovementioned cells were conventionally cultured in the RPMI 1640 medium (Gibco, USA) with 10% fetal bovine serum (FBS, Gibco, USA) in a saturated humidity incubator at 37°C, 5% CO_2_.

AFAP1-AS1 small interfering RNA (si-AFAP1-AS1) and disordered nonsense-negative sequence (si-NC), WISP1 overexpression vector (pc-WISP1) and empty vector (pc-NC), miR-195-5p mimic (mimic) and negative control (miR-NC), and miR-195-5p inhibitor (inhibitor) and negative control (anti-NC) were purchased from Shanghai Genepharma Co., Ltd. Cells at a logarithmic proliferative stage were inoculated into a 6-well plate at 5 × 10^6^ cells/cm^2^ that the confluence of cells at transfection was above 50%. The cells were transfected by using the Lipofectamine^TM^ 2000 liposome kit (Invitrogen, USA). After transfection for 6 h, the cells were replaced with fresh medium and cultured for 48 h.

### 2.3. Colony Formation Assay

Cells of each group (1 × 10^3^ cells/well) were cultured in 6-well plates. After 14 days of culture, the medium was discarded and cells were fixed with paraformaldehyde (4%) and then stained with crystal violet (0.5%). After drying, photos were taken with a microscope and the clone numbers were observed. A colony larger than 50 cells is counted as a clone. The experiment was repeated three times independently.

### 2.4. Wound-Healing Assay

After trypsin digestion, cells were cultured with 6-well plates at 5 × 10^5^ cells/ml. When the degree of cell fusion reached 100%, the cells were scratched at the bottom of the culture well with the tip of a 10 *μ*l pipettor. After continuous culture for 48 h, the scar width was measured and the healing rate was calculated.

Scratch healing rate (%) = (scratch width_0 h_−scratch width_48 h_)/scratch width_0 h_ × 100%. The experiment was conducted independently in triplicate.

### 2.5. Transwell Assay

An RPMI1640 medium without FBS was used to adjust the concentration of transfected cells to 5 × 10^4^ cells/ml. The upper chamber of Transwell was precoated with matrigel, and 100 *μ*l cell suspension was added. 500 *μ*l RPMI 1640 medium containing FBS was added to the lower chamber. After incubation for 48 h, 4% paraformaldehyde was fixed for 30 min and stained with 0.4% crystal violet. An inverted microscope was selected to perform the observation and counting. The experiment was repeated three times.

### 2.6. Dual-Luciferase Reporter Assay

ENCORI (the encyclopedia of RNA interactomes) database (http://starbase.sysu.edu.cn/index.php) predicted that continuous binding sites exist between AFAP1-AS1 and miR-195-5p nucleotide sequences. The TargetScan database (http://www.targetscan.org/vert_72/) shows that there are miR-195-5p binding sites in the WISP1 3′UTR region. Fragments of the wild type (wt) and mutant type (mut) of AFAP1-AS1 and WISP1 were constructed and integrated into the pGL3 vector to construct a report vector (AFAP1-AS1-wt, AFAP1-AS1-mut, WISP1-wt, and WISP1-mut), respectively. Cells were transfected with miR-195-5p mimic or miR-NC with wt or mut reporter vectors, respectively. Luciferase activity was measured after 48 h transfection. Each experiment was repeated 3 times.

### 2.7. RT-qPCR

Total RNA was extracted from tissue and cells by the Trizol reagent (Takara, Japan) and reverse transcribed into cDNA using the RT-PCR kit (Invitrogen, USA). RT-qPCR was performed with the SYBR Green kit (Takara, Japan) on ABI7500 Applied Biosystems. Primers are listed in Table 1. All data were calculated using the 2^−ΔΔCT^ method. Data were obtained from three independent experiments.

### 2.8. Western Blot Assay

RIPA lysate (Beyotime, China) was used to extract the total protein from the cells, and a BCA kit (Beyotime, China) was used to detect the protein concentration. SDS-PAGE was performed with 30 *μ*g protein per well. After electrophoresis, proteins were transferred to polyvinylidene fluoride (PVDF) membranes (Millipore, USA). Membranes were placed in 5% skimmed milk powder for sealing 1 h. Then, WISP1 and GAPDH antibodies were added, respectively. After incubation at 4°C overnight, horseradish peroxidase-labeled secondary antibody was incubated at room temperature for 1 h. A chemiluminescence reagent (Beyotime, China) was added to avoid light development, and exposure was taken by using a gel imaging system. The analysis was performed independently in triplicate.

### 2.9. Statistical Analysis

GraphPad Prism 6.0 was used for data analysis, and all data were expressed as mean ± standard deviation. The *t* test was used for comparison between the two groups. One-way analysis of variance was used for comparison between multiple groups. The significance level was *P* < 0.05.

## 3. Results

### 3.1. AFAP1-AS1 Expression Was Increased in CRC

**Figure 1:**
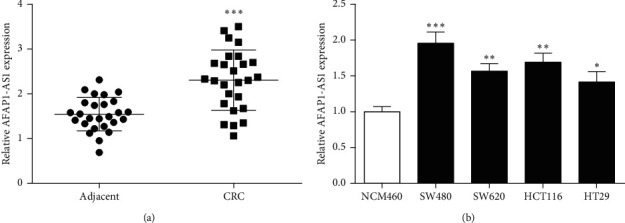
Expressions of AFAP1-AS1 in CRC. (a) Expression of AFAP1-AS1 in CRC tissues and adjacent tissues was detected by RT-qPCR. (b) Expression of AFAP1-AS1 in CRC cells was detected. ^*∗*^*P* < 0.05, ^*∗∗*^*P* < 0.01, and ^*∗∗∗*^*P* < 0.001, compared with adjacent or human normal colon cell lines NCM460.

As shown in Figure 1(a), AFAP1-AS1 expression was observably increased in CRC tissues versus paracancerous tissues. Similarly, AFAP1-AS1 expression in SW480, SW620, HCT116, and HT29 were also significantly increased versus NCM460 (Figure 1(b)). AFAP1-AS1 expression was the highest in SW480 cells, so SW480 cells were selected as the cell carrier for the subsequent experiments.

### 3.2. AFAP1-AS1 Knockdown Suppressed CRC Cell Malignant Behavior

**Figure 2:**
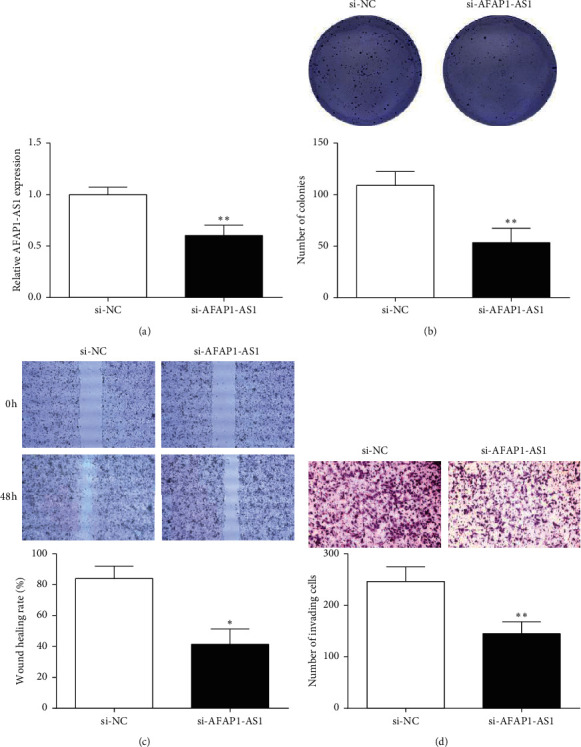
Effects of AFAP1-AS1 on CRC cell behavior. (a, b) The transfection efficiency of si-AFAP1-AS1 was detected by RT-qPCR. (b) Colony formation assay was conducted to evaluate the effect of si-AFAP1-AS1 on the proliferation of CRC cells (×200). (c) Wound-healing assay showed that si-AFAP1-AS1 inhibited migration of CRC cells (×200). (d) Effects of si-AFAP1-AS1 on the invasion of CRC cells were detected by Transwell assay (×200). ^*∗*^*P* < 0.05 and ^*∗∗*^*P* < 0.01, compared with the si-NC group.

To verify the role of AFAP1-AS1 in CRC, si-AFAP1-AS1 was transfected into SW480 cells, and AFAP1-AS1 expression was assessed by RT-qPCR. Results showed that AFAP1-AS1 expression of the si-AFAP1-AS1 group was obviously decreased versus si-NC (Figure 2(a)). The abovementioned results suggested si-AFAP1-AS1 has a good inhibitory efficiency and further experiments could be carried out.

After si-AFAP1-AS1 was transfected, the effects of AFAP1-AS1 inhibition on the malignant behavior of SW480 cells were verified by colony formation, wound-healing, and Transwell assay. Cell clone formation assay revealed the clone formation number of SW480 with si-AFAP1-AS1 was notably reduced versus si-NC (Figure 2(b)). This suggested that AFAP1-AS1 inhibition dramatically inhibits the proliferation of SW480 cells. The healing rate of the si-AFAP1-AS1 was memorably decreased versus si-NC at 48 h (Figure 2(c)). At the same time, the number of invading cells in si-AFAP1-AS1 was markedly reduced compared to si-NC (Figure 2(d)). We suggested that inhibition of AFAP1-AS1 expression hampered SW480 cell migration and invasion.

### 3.3. AFAP1-AS1 Directly Targeted miR-195-5p

**Figure 3:**
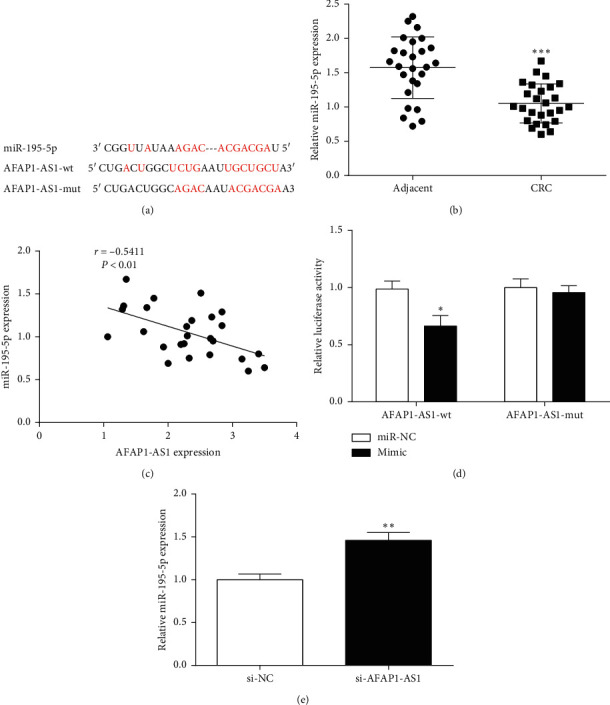
The relationship between AFAP1-AS1 and miR-195-5p. (a) Binding sites between AFAP1-AS1 and miR-195-5p were predicted by bioinformatics software. (b) The expression of miR-195-5p was decreased in CRC tissues. (c) Pearson correlation analysis revealed that a negative association existed between miR-195-5p and AFAP1-AS1 expression in CRC tissues. (d) Luciferase activity assay was performed to determine the relationship between AFAP1-AS1 and miR-195-5p. (e) The changes of miR-195-5p expression in CRC cells after AFAP1-AS1 knockdown. ^*∗*^*P* < 0.05, ^*∗∗*^*P* < 0.01, and ^*∗∗∗*^*P* < 0.001, compared with the adjacent, miR-NC, or si-NC group.

The existence of binding sites between AFAP1-AS1 and miR-195-5p was predicted through the Starbase database (Figure 3(a)). miR-195-5p expression in tissues was detected by RT-qPCR. miR-195-5p expression was found to decrease in CRC tissues compared with paracancerous tissues (Figure 3(b)). Meanwhile, Pearson's correlation analysis results found AFAP1-AS1 expression was negatively correlated with miR-195-5p expression in tissues (Figure 3(c).

AFAP1-AS1 and miR-195-5p's relationship was further explored through dual-luciferase assay. The results showed that luciferase activity could be significantly inhibited by miR-195-5p mimic and AFAP1-AS1-wt, but it had no change of miR-195-5p mimic and AFAP1-AS1-mut (Figure 3(d)). Additionally, the miR-195-5p expression was promoted after AFAP1-AS1 knockdown (Figure 3(e)). In conclusion, AFAP1-AS1 targeted miR-195-5p and negative regulate its expression in CRC.

### 3.4. WISP1 Was a Target of miR-195-5p

**Figure 4:**
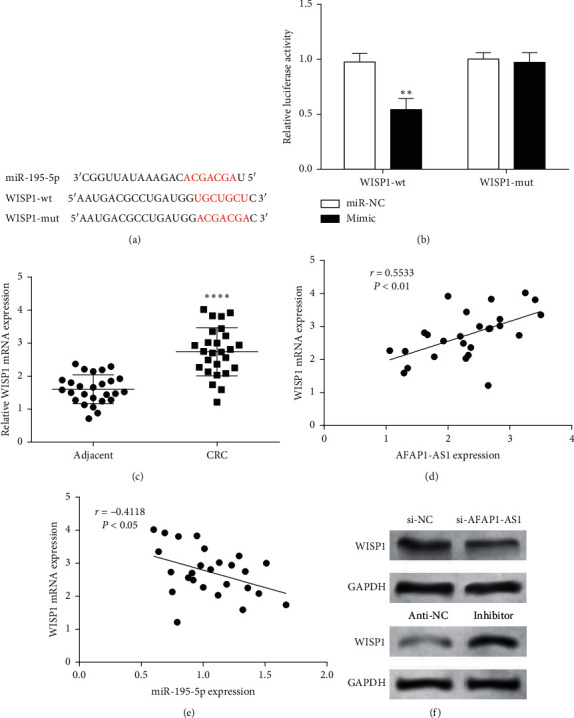
WISP1 was targeted by miR-195-5p. (a) TargetScan predicted the binding site between miR-195-5p and WISP1. (b) Dual-luciferase reporter gene assay determined WISP1 was targeted by miR-195-5p. (c) WISP1 was upregulated in CRC tissues. (d) Correlation analysis between AFAP1-AS1 and WISP1 expression in CRC tissues. (e) miR-195-5p expression was negatively correlated with WISP1 expression in CRC tissues. (f) Effects of AFAP1-AS1 and miR-195-5p on WISP1 protein expression. ^*∗∗*^*P* < 0.01 and ^*∗∗∗∗*^*P* < 0.0001, compared with the adjacent or miR-NC group.

According to TargetScan database analysis, binding sequences of miR-195-5p and WISP1 were found (Figure 4(a)). Whether WISP1 binds to miR-195-5p, detection of luciferase activity was performed through a dual-luciferase experiment. Compared with mutant WISP1-mut, luciferase activity of WISP1-wt was markedly reduced in the presence of mimic (Figure 4(b)). The results showed that miR-195-5p could specifically bind WISP1.

WISP1 mRNA expression in CRC and its adjacent tissues was detected by RT-qPCR, and then, Pearson's correlation analysis was used for correlation analysis. Compared with adjacent tissues, WISP1 mRNA expression was notably increased in CRC tissues (Figure 4(c)). Moreover, WISP1 mRNA expression was negatively correlated with miR-195-5p expression (Figure 4(d)), while the expression of AFAP1-AS1 and WISP1 mRNA has a positive correlation (Figure 4(e)). Western blotting results showed that AFAP1-AS1 knockdown could decrease WISP1 expression, while the miR-195-5p inhibitor could increase WISP1 expression (Figure 4(f)). Thus, it was speculated that WISP1 is a direct target of miR-195-5p and is negatively regulated by miR-195-5p and positively regulated by AFAP1-AS1.

### 3.5. AFAP1-AS1/miR-195-5p/WISP1 Facilitated CRC Progression

**Figure 5:**
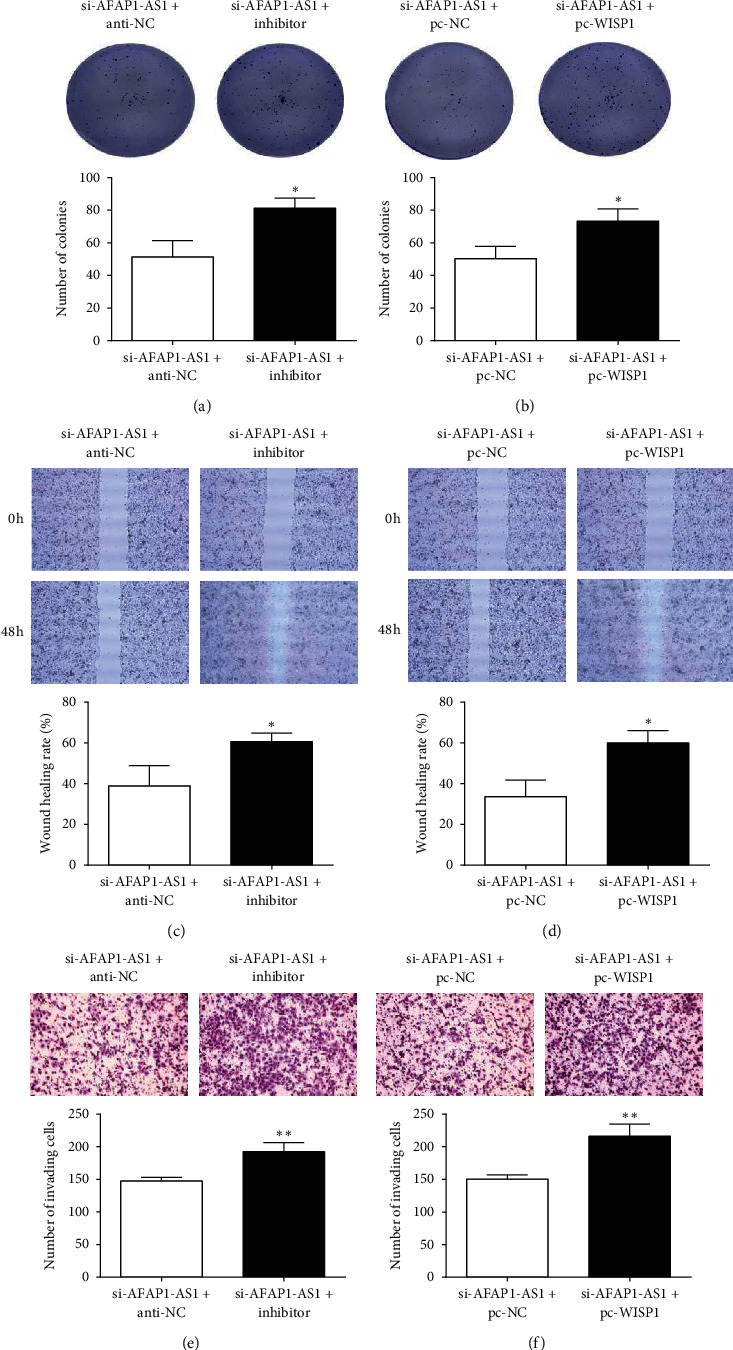
WISP1 overexpression or miR-195-5p inhibitor reverses the function of AFAP1-AS1 knockdown on the malignant behavior of CRC cell. (a, b) The proliferation of CRC cells was measured by colony formation assay (×200). (c, d) The migration ability of CRC cells was examined using a wound-healing assay (×200). (e, f) Transwell assay was conducted to assess the invasion of CRC cells (×200). ^*∗*^*P* < 0.05 and ^*∗∗*^*P* < 0.01, compared with the si-AFAP1-AS1 + anti-NC or si-AFAP1-AS1 + pc-NC group.

To further confirm the function of the AFAP1-AS1/miR-195-5p/WISP1 pathway in CRC, we performed the rescue experiment by colony formation, wound-healing, and Transwell assays. WISP1 overexpression or miR-195-5p inhibition rescued the cell malignant behavior induced by AFAP1-AS1 knockdown (Figure 5). Collectively, results verified that AFAP1-AS1 facilitated CRC progression through modulating miR-195-5p/WISP1 axis expression.

## 4. Discussion

The pathogenesis of CRC is still not very clear, and its occurrence and development involve the expression disorder or functional abnormality of several genes [14]. How to early diagnose and predict the poor prognosis of CRC and take intervention measures as early as possible is very important to reduce the mortality of patients. Hence, it has great clinical significance in finding key molecules involved in the occurrence and development of CRC and in searching for biomarkers for early diagnosis and treatment of CRC.

LncRNAs play a vital role in tumor growth and can be used as molecular targets for the regulation of tumor progression [15]. Studies have shown that AFAP1-AS1 expression is increased, and its knockdown inhibits osteosarcoma progression by regulating miR-497/IGF1R [16]. In oral squamous cell carcinoma (OSCC), AFAP1-AS1 expression is enhanced and its inhibition can suppress the proliferation, migration, and invasion, while promoting cell apoptosis via affecting the miR-145/HOXA1 axis [17]. This study showed that AFAP1-AS1 expression was enhanced in CRC, suggesting that AFAP1-AS1 might involve in CRC occurrence and development. After transfection of si-AFAP1-AS1 into CRC cells, the cell survival, the number of clone formation, migration distance, and invaded cells number were decreased, which is consistent with previous reports [9–11]. However, research on the regulatory mechanism of AFAP1-AS1 in CRC cell biological behaviors is scarce.

In order to further explore the molecular mechanism by which AFAP1-AS1 affects CRC occurrence and development, this study confirmed that AFAP1-AS1 negatively regulates miR-195-5p expression by double-luciferase and RT-qPCR assays. Studies have shown that miR-195-5p overexpression inhibits the cell biological behaviors of cervical cancer, lung cancer, and other tumor cells and can be used as a potential molecular target for tumor therapy [18, 19]. Similarly, miR-195-5p also acts as a tumor suppressor in CRC [12]. More importantly, this study showed that inhibition of miR-195-5p reduced the inhibitory effect of AFAP1-AS1 knockdown on the proliferation, migration, and invasion of CRC cells, suggesting that AFAP1-AS1 affected the cell biological behaviors of CRC by regulating the expression of miR-195-5p.

In tumors, miRNA usually affects the cell's biological behaviors by targeting downstream target genes [20]. In this study, we found the binding site of miR-195-5p and WISP1 was existed according to bioinformatics analysis. WISP1 is a secreted extracellular-matrix-related protein and a member of the cellular communication network (CCN) growth factor family [21]. CCN protein is abnormally expressed in tumors [22]. As a member of this family, WISP1 has many developmental functions and may be related to tumorigenesis [23]. Thus, whether AFAP1-AS1 regulates proliferation, migration, and invasion of CRC cells through the regulation of WISP1 was further explored. In our double-luciferase report, the target relationship of miR-195-5p and WISP1 was confirmed. Additionally, AFAP1-AS1 expression has a positive correlation with WISP1 mRNA expression in CRC tissues. Moreover, AFAP1-AS1 inhibition could reduce WISP1 expression while miR-195-5p inhibition could enhance WISP1 expression. Moreover, WISP1 overexpression can offset the inhibitory effect of AFAP1-AS1 knockdown on CRC cells proliferation, migration, and invasion. We speculated that AFAP1-AS1 is involved in CRC progression, and the mechanism may be related to the regulation of the miR-195-5p/WISP1 pathway.

## 5. Conclusions

In conclusion, it was found that AFAP1-AS1 expression was increased in CRC. Further studies verified that AFAP1-AS1 knockdown inhibited the proliferation, invasion, and migration of CRC cells. Related mechanism experiments suggested that AFAP1-AS1 facilitated CRC progression by regulating the miR-195-5p/WISP1 axis. This study provides new insights into CRC pathogenesis and contributes to CRC diagnosis and treatment. However, there are still some shortcomings in this study. The study was limited to a small tissue sample, one cell line in functional experiments, and in vitro experiments. Further confirmation of the abovementioned mechanisms is needed with larger samples (relationship of AFAP1-AS1 expression with clinicopathological characteristics and prognosis), two or more cell lines, and in vivo experiments. Also, more experimental exploration is needed to confirm whether it can be used in clinical diagnosis and targeted therapy.

## Figures and Tables

**Table 1:** Primer sequences for real-time fluorescence quantification PCR.

Gene name	Primer sequences (5′-3′)
GAPDH	F AAGGTGAAGGTCGGAGTCAA
	R AATGAAGGGGTCATTGATGG

U6	F GCTTCGGCAGCACATATACTAAAAT
	R CGCTTCACGAATTTGCGTGTCAT

AFAP1-AS1	F AGCCTTGGTGAGCAATAGGT
	R GGTATGAAGGGTGTGGGTGA

miR-195-5p	F GGGGTAGCAGCACAGAAAT
	R CAGTGCGTGTCGTGGAGT

WISP1	F GAAGCAGTCAGCCCTTATG
	R CTTGGGTGTAGTCCAGAAC

## Data Availability

The datasets used and/or analyzed during the present study are available from the corresponding author on reasonable request.

## References

[1] Bray F., Ferlay J., Soerjomataram I., Siegel R. L., Torre L. A., Jemal A. (2018). Global cancer statistics 2018: GLOBOCAN estimates of incidence and mortality worldwide for 36 cancers in 185 countries. *CA: A Cancer Journal for Clinicians*.

[2] Mauri G., Sartore‐Bianchi A., Russo A. G., Marsoni S., Bardelli A., Siena S. (2019). Early‐onset colorectal cancer in young individuals. *Molecular Oncology*.

[3] Oh H.-H., Joo Y.-E. (2020). Novel biomarkers for the diagnosis and prognosis of colorectal cancer. *Intestinal Research*.

[4] Aran V., Victorino A. P., Thuler L. C., Ferreira C. G. (2016). Colorectal cancer: epidemiology, disease mechanisms and interventions to reduce onset and mortality. *Clinical Colorectal Cancer*.

[5] Chan J., Tay Y. (2018). Noncoding RNA:RNA regulatory networks in cancer. *International Journal of Molecular Sciences*.

[6] Poursheikhani A., Abbaszadegan M. R., Kerachian M. A. (2020). Mechanisms of long non‐coding RNA function in colorectal cancer tumorigenesis. *Asia-Pacific Journal of Clinical Oncology*.

[7] Zhang F., Li J., Xiao H., Zou Y., Liu Y., Huang W. (2018). AFAP1-AS1: a novel oncogenic long non-coding RNA in human cancers. *Cell Proliferation*.

[8] Ji D., Zhong X., Jiang X. (2018). The role of long non-coding RNA AFAP1-AS1 in human malignant tumors. *Pathology—Research and Practice*.

[9] Han X., Wang L., Ning Y., Li S., Wang Z. (2016). Long non-coding RNA AFAP1-AS1 facilitates tumor growth and promotes metastasis in colorectal cancer. *Biological Research*.

[10] Feng W., Ni H., Feng S., Min L., Lin C. (2016). Overexpression of lncRNA AFAP1-AS1 correlates with poor prognosis and promotes tumorigenesis in colorectal cancer. *Biomedicine & Pharmacotherapy = Biomedecine & Pharmacotherapie*.

[11] Tang J., Zhong G., Wu J., Chen H., Jia Y. (2018). Long noncoding RNA AFAP1-AS1 facilitates tumor growth through enhancer of zeste homolog 2 in colorectal cancer. *American Journal of Cancer Research*.

[12] Sun M., Song H., Wang S. (2017). Integrated analysis identifies microRNA-195 as a suppressor of Hippo-YAP pathway in colorectal cancer. *Journal of Hematology & Oncology*.

[13] Wu J., Long Z., Cai H. (2016). High expression of WISP1 in colon cancer is associated with apoptosis, invasion and poor prognosis. *Oncotarget*.

[14] Palhares D. M. F., Reis Figueiredo R., Marchetti K. R. (2020). Stereotactic radiotherapy (SRT) in oligometastatic (OM) colorectal cancer (CRC): can we improve systemic therapy (ST) free interval?. *Journal of Clinical Oncology*.

[15] Lin Y.-H. (2020). Crosstalk of lncRNA and cellular metabolism and their regulatory mechanism in cancer. *International Journal of Molecular Sciences*.

[16] Fei D., Zhang X., Lu Y., Tan L., Xu M., Zhang Y (2020). Long noncoding RNA AFAP1-AS1 promotes osteosarcoma progression by regulating miR-497/IGF1R axis. *American Journal of Translational Research*.

[17] Li M., Yu D., Li Z., Zhao C., Su C., Ning J. (2020). Long non‑coding RNA AFAP1‑AS1 facilitates the growth and invasiveness of oral squamous cell carcinoma by regulating the miR‑145/HOXA1 axis. *Oncology Reports*.

[18] Long Z., Wang Y. (2020). miR-195-5p suppresses lung cancer cell proliferation, migration, and invasion via FOXK1. *Technology in Cancer Research Treatment*.

[19] Liu X., Zhou Y., Ning Y. E., Gu H., Tong Y., Wang N. (2020). MiR-195-5p inhibits malignant progression of cervical cancer by targeting YAP1. *OncoTargets and Therapy*.

[20] Nikitina E. G., Urazova L. N., Stegny V. N. (2012). MicroRNAs and human cancer. *Experimental Oncology*.

[21] Maiese K. (2014). WISP1: clinical insights for a proliferative and restorative member of the CCN family. *Current Neurovascular Research*.

[22] Dhar A., Ray A. (2010). The CCN family proteins in carcinogenesis. *Experimental Oncology*.

[23] Gurbuz I., Ruth C.-E. (2015). CCN4/WISP1 (WNT1 inducible signaling pathway protein 1): a focus on its role in cancer. *The International Journal of Biochemistry & Cell Biology*.

